# Evaluation of novel X-ray protective eyewear in reducing the eye dose to interventional radiology physicians

**DOI:** 10.1093/jrr/rrab014

**Published:** 2021-04-12

**Authors:** Mime Endo, Yoshihiro Haga, Masahiro Sota, Akiko Tanaka, Kazuki Otomo, Yuuki Murabayashi, Mitsuya Abe, Yuji Kaga, Yohei Inaba, Msatoshi Suzuki, Taiichiro Meguro, Koichi Chida

**Affiliations:** 1 Department of Radiological Technology, Tohoku University Graduate School of Medicine , 2-1 Seiryo, Aoba, Sendai 980-8575, Japan; 2 Department of Radiology, Sendai Kousei Hospital , Hirosemachi 4-15, Aobaku, Sendai 980-0873, Japan; 3 Department of Cardiovascular Medicine, Sendai Kousei Hospital , Hirosemachi 4-15, Aobaku, Sendai 980-0873, Japan; 4 Division of Disaster Medicine, International Research Institute of Disaster Science, Tohoku University , 6-6-4 Aoba, Sendai 980-8579, Japan

**Keywords:** radiation safety, eye lens dose, interventional radiology (IVR), fluoroscopically guided procedures, radiation disaster medicine, 3 mm dose equivalent [Hp(3)], Pb glasses (lead eyewear), X-ray fluoroscopy, disaster medicine

## Abstract

The new recommendation of the International Commission on Radiological Protection for occupational eye dose is an equivalent dose limit to the eye of 20 mSv year ^–1^ , averaged over a 5-year period. This recommendation is a drastic reduction from the previous limit of 150 mSv year ^–1^ . Hence, it is important to protect physicians’ eyes from X-ray radiation. Particularly in interventional radiology (IVR) procedures, many physicians use protective lead (Pb) glasses to reduce their occupational exposure. This study assessed the shielding effects of novel 0.07 mm Pb glasses.

The novel glasses (XR-700) have Pb–acrylic lens molded in three dimensions. We studied the novel type of 0.07 mm Pb glasses over a period of seven consecutive months. The eye dose occupational radiation exposure of seven IVR physicians was evaluated during various procedures. All IVR physicians wore eye dosimeters (DOSIRIS™) close to the left side of the left eye. To calculate the shielding effects of the glasses, this same type of eye dosimeter was worn both inside and outside of the Pb lenses.

The average shielding effect of the novel glasses across the seven physicians was 61.4%. Our results suggest an improved shielding effect for IVR physicians that use these glasses. No physician complained that the new glasses were uncomfortable; therefore comfort is not a problem.

The lightweight glasses were acceptable to IVR physicians, who often must perform long procedures. Thus, the novel glasses are comfortable and reasonably protective. Based on the results of this study, we recommend that IVR physicians use these novel 0.07 mm Pb glasses to reduce their exposure.

## INTRODUCTION

 Interventional radiology (IVR) procedures, which can substantially benefit patients, can also injure both patients and physicians due to exposure to X-ray radiation [ 1–9 ]. Thus, radiation protection of patients and physicians in IVR is very important [ 10–18 ]. 

 The new recommendation of the International Commission on Radiological Protection (ICRP) for occupational eye dose is an equivalent dose limit to the eye of 20 mSv year ^–1^ , averaged over a 5-year period, with no single year exceeding 50 mSv [ 19 , 20 ]. This recommendation is a drastic reduction from the previous limit of 150 mSv year ^–1^ . Hence, it has become more important than ever to evaluate the occupational exposure of IVR physicians and protect their eyes from X-rays using glasses with lead (Pb)-infused lenses [ 21–28 ]. 

 Because IVR involves procedures of long duration, lightweight Pb glasses (i.e. 0.07 mm Pb-equivalent) are preferable for physicians. Thus, 0.07 mm Pb glasses have gradually become widely used in IVR procedures. While such glasses are lightweight and comfortable, the version currently on the market reduces X-rays in IVR procedures by only ~50–60% [ 29–31 ]. We believe that this shielding effect is not sufficient and that more effective eye protection is needed. 

 Although the radiation shielding effect of 0.75 mm Pb-equivalent glasses is excellent, such glasses are heavy and uncomfortable, especially during long procedures [ 32 ]. 

Recently, an improved version of X-ray-protective 0.07 mm Pb eyewear has been developed. In this study, we evaluated the X-ray shielding effects of these novel glasses in an IVR clinical setting.

## MATERIALS AND METHODS

### Novel 0.07 mm Pb-equivalent eyewear

The novel glasses (XR-700) have Pb–acrylic lens molded in three dimensions; thus, we can expect that scattered radiation from the sides and underneath will be cut drastically. The novel glasses are lightweight, with a mass of 42 g. For improved fit, and thus improved protection by minimizing the gaps between the Pb–acrylic lenses and the face, the glasses offer two ways in which they can be adjusted to the facial shape of the physician: (i) the gap between the nose pads has an adjustable width, allowing the bridge to be adjusted by up to ~5 mm; and (ii) the angle of the sides (temples) of the glasses can be adjusted in four steps, with a change of 9° for each step.

### Dosimetry

**Fig. 1.**
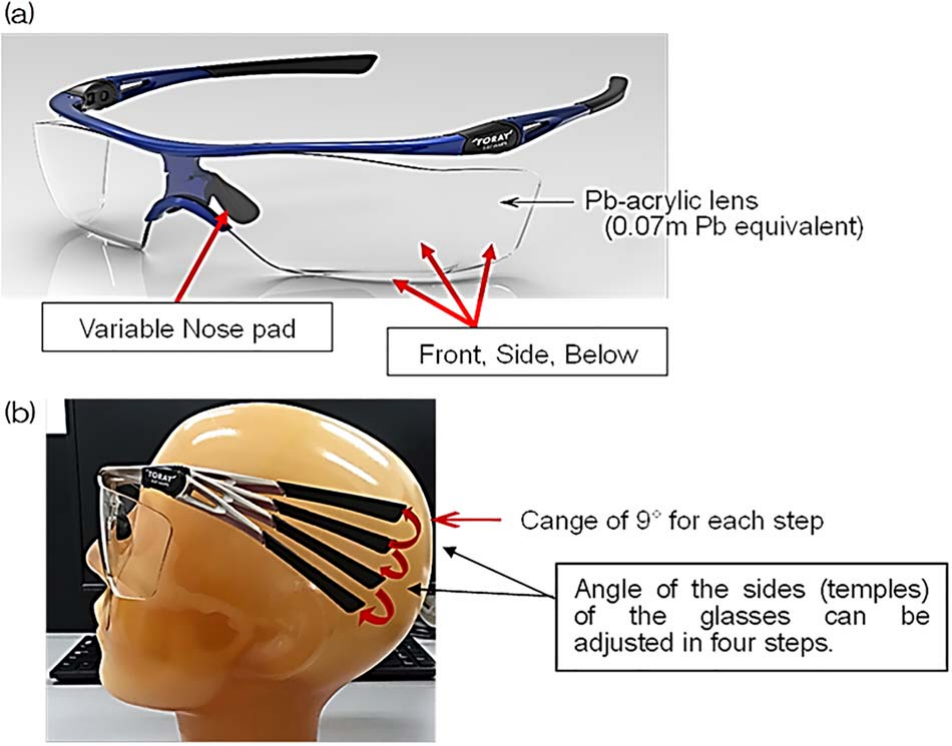
Novel protective eyewear that shields the eyes from X-ray radiation. (a) Details of the new 0.07 mm Pb protective glasses. • The lenses of the novel glasses are molded three-dimensionally, so that they can block scattered radiation not only from the front but also from the sides and below. • The nose pad and side (temple) of the spectacles can be adjusted. These light and comfortable 0.07 mm Pb protective glasses have been adapted to reduce the burden on the operator during a long procedure. (b) Overview of how the angle of the temple can be adjusted. Four adjustments can be made in steps, with a change of 9° for each step.

 We used digital angiography X-ray systems with a flat-panel detector (Infinix Celeve-i, CANON, Japan) for all procedures. We studied the novel type of 0.07 mm Pb glasses ( Fig. 1 ) over a period of seven consecutive months, during which time doses were monitored over 1-month intervals. In our institution, the eye dose occupational radiation exposure of seven IVR physicians was evaluated during various procedures: coronary angiography, percutaneous coronary intervention, percutaneous peripheral intervention, pacemaker implantation and catheter ablation. During these procedures, the IVR physicians wore the novel glasses. 

 During all procedures, the physicians were typically positioned close to the right side of the patient and used a ceiling protection plate (0.5 mm Pb) if one was available. The methods used to evaluate the eye radiation dose have been described previously [ 29 ]. Briefly, all IVR physicians wore eye dosimeters (DOSIRIS™) close to the left side of the left eye; these measure the 3 mm dose equivalent, Hp(3). To calculate the shielding effects of the glasses, this same type of eye dosimeter was worn both inside and outside of the Pb lenses, as in our previous study [ 29 ]. Using the resulting outside (D _out_ ) and inside (D _in_ ) doses, we calculated the shielding effect of the glasses as follows: 

 Shielding effect = ( *D*_out_ – *D*_in_ )/ *D*_out_ × 100% 

 We also determined the estimated annual eye dose (EAED) as follows:

$$\mathrm{EAED}\ \left(\mathrm{mSv}\ \mathrm{year}^{-1}\right)=\mathrm{measured}\ \mathrm{monthly}\ \mathrm{dose}\times 12 $$

## RESULTS

Table 1 lists details about the procedures conducted by the seven physicians. No physician complained that the new glasses were uncomfortable, thus comfort is not a problem. 

**Table 1:** Details of the procedures conducted by each physician

Physician no.	Number of procedures	Fluoroscopy time (min)	Used size of the Pb glasses
1	20.9 ± 5.2	508.8 ± 169.1	Small
2	21.9 ± 3.4	247.3 ± 83.8	Large
3	10.4 ± 3.6	120.5 ± 44.6	Large
4	19.4 ± 4.3	218.6 ± 55.0	Large
5	12.7 ± 3.0	183.7 ± 35.1	Regular
6	22.0 ± 6.1	254.6 ± 89.4	Regular
7	10.9 ± 3.5	136.3 ± 73.2	Regular
Average ± SD	15.1 ± 6.2	238.5 ± 120.2	

Results are presented as the mean ± standard deviation (SD) of the monthly averages

**Table 2:** Summary of the results of our 7-month study

Physician no.	Inside dose [Hp(3)] (mSv)	Outside dose [Hp(3)] (mSv)	Shielding effect (%)
1	1.04 ± 0.17	2.64 ± 0.3	60.9 ± 2.43
2	0.80 ± 0.38	1.97 ± 0.85	59.61 ± 2.93
3	0.17 ± 0.09	0.44 ± 0.28	60.13 ± 5.12
4	0.59 ± 0.18	1.61 ± 0.41	63.36 ± 3.23
5	0.50 ± 0.22	1.31 ± 0.60	58.85 ± 4.12
6	1.04 ± 0.33	2.76 ± 0.93	60.81 ± 1.06
7	0.45 ± 0.2	1.14 ± 0.51	61.83 ± 0.77
Average ± SD	0.66 ± 0.30	1.70 ± 0.77	61.4 ± 1.91

Results are presented as the mean ± standard deviation (SD) of the monthly averages.

Table 2 summarizes the results of our study. The average shielding effect of the novel glasses across the seven physicians was 61.4%. The glasses come in three sizes (small, regular and large), and it seems that the shielding effect did not significantly differ with the size. 

Table 3 lists the estimated annual equivalent dose to the lens of the eye across all physicians while using the novel glasses. The radiation doses (mean ± standard deviation) inside and outside of the novel glasses were 7.9 ± 3.6 and 20.4 ± 9.2 mSv year ^–1^ , respectively. 

**Table 3:** Estimated annual dose to the lens of the eye

Physician no.	Inside dose [Hp(3)] (mSv)	Outside dose [Hp(3)] (mSv)
1	12.5 ± 2.0	31.7 ± 3.6
2	9.6 ± 4.6	23.6 ± 10.2
3	2.0 ± 1.1	5.3 ± 3.4
4	7.1 ± 2.2	19.3 ± 4.9
5	6.0 ± 2.6	15.7 ± 7.2
6	12.5 ± 4.0	33.1 ± 11.2
7	5.4 ± 2.4	13.7 ± 6.1
Average ± SD	7.9 ± 3.9	20.3 ± 10.0

**Fig. 2.**
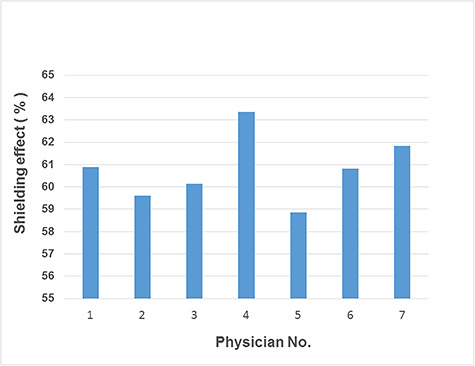
Seven-month average of the shielding effect for each physician.

**Fig. 3.**
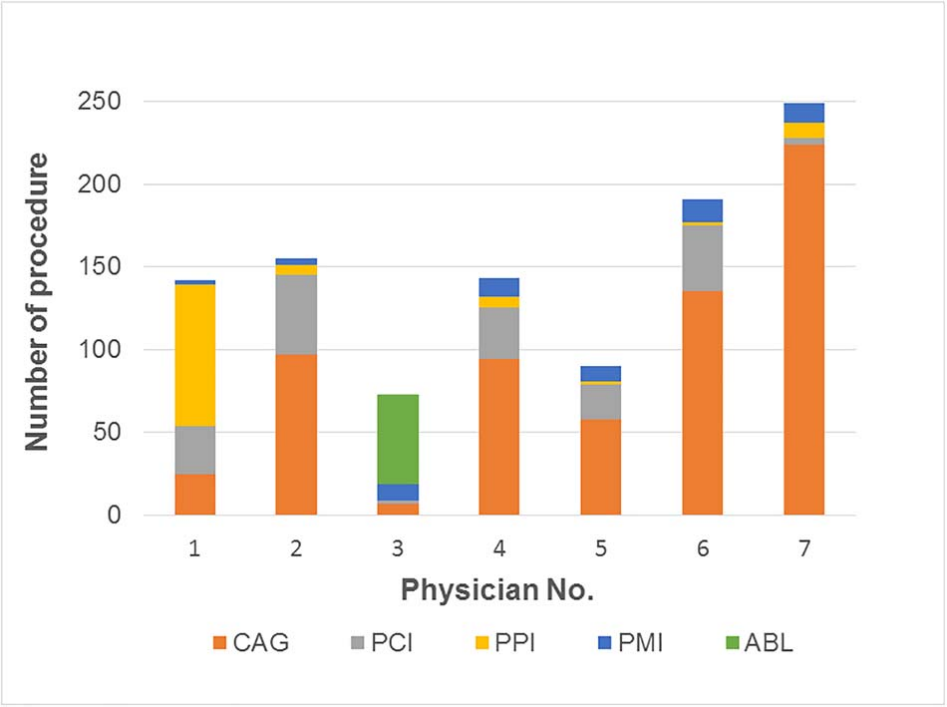
Numbers and types of procedures performed by each physician. CAG, coronary angiography; PCI, percutaneous coronary intervention; PPI, percutaneous peripheral intervention; PMI, pacemaker implantation; ABL, catheter ablation.

**Fig. 4.**
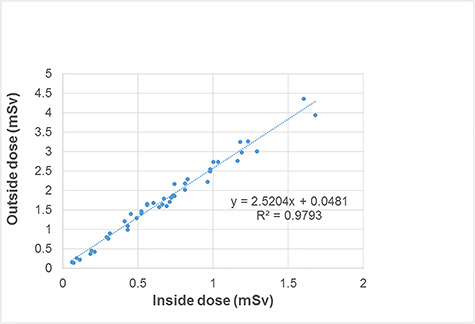
Differences between the doses measured near the eye inside and outside the glasses.

Figure 2 shows a bar graph of the average shielding effect of the novel glasses, and Figure 3 shows the numbers and types of procedure for each physician; most of them were for coronary angiography. Figure 4 shows the correlations between the doses (mSv month ^−1^ ) inside and outside the novel glasses, which were significant ( *R* ^2^ = 0.98). 

## DISCUSSION

 Although reports have been published on the basic effects of radiation protection products [ 33–43 ], few studies have evaluated the shielding effects of Pb glasses in a clinical setting, such as IVR [ 29–31 ]. Prevention of occupational eye radiation dose is important, particularly for IVR physicians; thus, Pb glasses that provide better shielding are required. Although heavy Pb glasses (i.e. 0.75 mm Pb) have such an effect, they are uncomfortable and thus physicians might not tolerate wearing them for long procedures. Thus the development of new, light (0.07 mm Pb) eyewear is desirable. 

 Now, such eyewear has been developed, and we performed a clinical study of their use during IVR procedures. For the first time, we present occupational eye dose data of seven physicians over 7 months and can report the shielding effects of these glasses. Previous versions of these glasses could block ~50–60% of the X-ray dose in clinical settings [ 29–31 ]. We found that the new versions block ~61.4% ( Table 2 ), a slight improvement, although it is not clear whether there is a statistically significant difference. 

One likely reason for this improvement is that the scattered rays coming from below may be shielded by the new glasses as the gap between the lenses and the face is reduced through adjustments at the nose and at the temples. Moreover, there are three different sizes of glasses, possibly further improving fit. These results indicate that the novel glasses are useful for reducing eye exposure.

 Because of the strong correlation ( *R* ^2^ = 0.979) between the measurements made inside and outside of the novel glasses, it may be possible to estimate the inner dose from one dosimeter placed outside of them. However, in our dosimeter test setting, the shielding effect may contribute to a reduction in radiation exposures, mainly in the AP direction, because, the dosimeters are attached on the front and back of the glass. In actual exposure scenarios (i.e. in a clinical setting), scattered radiation can enter the eyes from above, from below and from the side, as well as from the front. Therefore, more research may be required in actual exposure scenarios in order to estimate the eye doses more accurately. 

 The shielding effect of the glasses, the type and number of procedures for each physician and the fluoroscopic time are shown in Figs 2 and 3 and Table 1 , respectively. The results show that the shielding effect did not differ greatly with the type and number of procedures or the fluoroscopic time. 

 In our study, the occupational EAED of the IVR physicians was lower than the new maximum allowable radiation limit (20 mSv year ^–1^ ). 

 Finally, like the previous versions, the new glasses are also light and comfortable. However, a previous study (Monte Carlo simulation method, i.e. computer-based calculation) reported a shielding effect of 74% for ‘wrap-around’ 0.07 mm Pb glasses [ 36 ]. The novel 0.07 mm Pb glasses tested in this clinical study are of a ‘wrap-around’ design, but the shielding effect was lower than projected by the Monte Carlo simulation. This was probably because the glasses are not fully ‘wrap-around’ when in use in the clinical IVR setting because there is a small gap between the face and the glasses. In addition, the value of the shielding effect might be affected by variations in the direction, position and angle of the physician’s head during the procedure. Thus, the version we tested requires further improvement to achieve a fully ‘wrap-around’ design in clinical settings. 

In summary, it is important to protect physicians’ eyes from X-ray radiation. Particularly in IVR procedures, many physicians use protective Pb glasses to reduce their occupational exposure. However, the shielding effects of Pb glasses depend on their specific features, and the impact of these is unclear in clinical settings. This study assessed the shielding effects of novel 0.07 mm Pb glasses worn by seven physicians in IVR laboratories for seven consecutive months. The average shielding effect was 61.4%. The new, improved 0.07 mm Pb glasses are as comfortable as the previous version. Because IVR procedures are typically of long duration, we recommend that physicians wear lightweight glasses. We particularly recommend that IVR physicians use the novel 0.07 mm Pb glasses to reduce their X-ray exposure.

This was an initial study of the novel 0.07 mm Pb glasses. Further investigation and statistical analysis are required based on a controlled comparison study, such as a multiinstitute evaluation over a long duration (a full year).

## CONCLUSIONS

We performed a clinical study of the physician eye dose and shielding effect of novel 0.07 mm Pb glasses during cardiac IVR procedures. The average shielding effect of the glasses was >60%. Our results imply some improvement in shielding of the eyes of IVR physicians that use these glasses. The lightweight glasses were found acceptable by IVR physicians, who often must perform long procedures. Thus, the novel glasses are comfortable and reasonably protective. Based on the results of this study, we recommend that IVR physicians use these novel 0.07 mm Pb glasses to reduce their exposure.
